# Patient Safety Culture, Second Victim Experience, and Post‐Traumatic Growth Among Hospital Nurses: A Cross‐Sectional Study

**DOI:** 10.1002/nop2.70623

**Published:** 2026-06-05

**Authors:** Hamidreza Siavashi, Ali Safdari, Maryam Farhadian, Maryam Maddineshat

**Affiliations:** ^1^ Student Research Committee Hamadan University of Medical Sciences Hamadan Iran; ^2^ Department of Nursing and Midwifery, School of Nursing Iran University of Medical Sciences Tehran Iran; ^3^ Student Research Committee Semnan University of Medical Sciences Semnan Iran; ^4^ Research Center for Health Sciences, Health Sciences and Technology Research Institute Hamadan University of Medical Sciences Hamadan Iran; ^5^ Department of Biostatistics, School of Public Health Hamadan University of Medical Sciences Hamadan Iran; ^6^ Chronic Diseases (Home Care) Research Center, Institute of Cancer, Hamadan University of Medical Sciences Hamadan Iran; ^7^ Department of Nursing, School of Malayer Medical Sciences Hamadan University of Medical Sciences Hamadan Iran

**Keywords:** emotional resilience, nursing, post‐traumatic growth, safety culture, second victim experience

## Abstract

**Aim:**

This study examined the relationships between patient safety culture, second victim experience, and post‐traumatic growth among nurses in Iran.

**Design:**

A cross‐sectional descriptive design was employed.

**Methods:**

A total of 435 nurses from various hospitals participated. Validated instruments were utilised to measure patient safety culture, second victim experience, and post‐traumatic growth. Data were analysed using one‐way ANOVA, independent samples t‐tests, Pearson's correlation, and multiple linear regression.

**Results:**

Mean scores were as follows: patient safety culture, 132.31 ± 16.12; second victim experience, 111.72 ± 19.47; and post‐traumatic growth, 59.05 ± 21.96. Patient safety culture was negatively correlated with second victim experience (*r* = −0.303, *p* < 0.001) and positively correlated with post‐traumatic growth (*r* = 0.330, *p* < 0.001). Female nurses reported higher second victim experience scores than males (*B* = 4.28, *p* = 0.026, 95% CI [0.50, 8.07]), and those working longer hours (> 30 h per week) also exhibited higher second victim experience scores (*B* = 14.25, *p* < 0.001, 95% CI [6.40, 22.11]). Regression analysis indicated that patient safety culture was associated with lower levels of second victim experience (*B* = −0.37, *p* < 0.001, 95% CI [−0.49, −0.26]); however, second victim experience did not directly predict post‐traumatic growth. The final model explained 13% of the variance in second victim experience.

**Conclusion:**

A stronger patient safety culture was associated with lower levels of second victim experiences and positively associated with post‐traumatic growth. Female nurses and those working longer hours reported higher levels of second victim experience. The lack of a direct association between second victim experience and post‐traumatic growth indicates that additional factors may shape this relationship. These findings highlight the importance of culturally sensitive safety initiatives and targeted support strategies for vulnerable groups. Longitudinal research is recommended to further clarify the temporal dynamics among these variables.

**Reporting Method:**

This study adhered to the STROBE reporting guidelines for cross‐sectional research.

**Patient or Public Contribution:**

None. This study did not involve patients or the public as it focused exclusively on nurses' self‐reported professional experiences.

## 1 Introduction

Medical errors constitute a significant source of preventable harm worldwide, contributing to an estimated 134 million adverse events annually in low‐ and middle‐income countries and leading to substantial morbidity and mortality (Mathebula et al. 2022). Nurses, who frequently work under high pressure and heavy workloads, are particularly susceptible to such errors (Foji et al. 2023). The impact of medical errors extends beyond patients; healthcare workers involved in these events—commonly referred to as “second victims”—often experience guilt, anxiety, shame, and self‐doubt. These emotional repercussions may persist over time (Choi et al. 2021; Foji et al. 2023), causing personal distress and increasing the likelihood of subsequent errors, thereby affecting both caregiver well‐being and patient safety (Busch et al. 2021). Although the prevalence and significance of second victim experiences are increasingly acknowledged across healthcare systems (Choi et al. 2021), the interplay between second victim experience, patient safety culture, and post‐traumatic growth (PTG) remains insufficiently understood. This gap is especially evident in low‐ and middle‐income countries such as Iran, where cultural and organisational factors may uniquely shape these relationships (Chen et al. 2021).

A strong patient safety culture plays a vital role in reducing medical errors and mitigating their emotional impact on healthcare providers. Core components—including open communication, non‐punitive responses to mistakes, and accessible support systems—encourage staff to report errors without fear of blame. Such environments promote transparency and continuous learning, potentially reducing adverse events while supporting the psychological well‐being of healthcare workers (Li et al. 2024). In nursing, particularly in demanding and multitasking settings, a supportive safety culture has been associated with higher job satisfaction, stronger professional commitment, and lower error rates. Evidence from Iran indicates that factors such as nurses' engagement, workload, and recovery from mistakes shape their perceptions of safety culture, while leadership behaviours such as coaching enhance error‐reporting willingness and strengthen safety culture overall (Chen et al. 2021; Istrate et al. 2025). Beyond preventing immediate errors, patient safety culture may also foster resilience and PTG among healthcare providers. PTG refers to positive psychological changes that may follow adverse or traumatic events, including involvement in medical errors. Studies suggest that environments characterised by teamwork, non‐punitive responses, and ongoing learning help providers rebuild confidence, reduce burnout, and experience personal growth after challenging incidents (Shomalinasab et al. 2023; Talebi et al. 2021). Given the substantial workload pressures faced by Iranian nurses (Foji et al. 2023; Matin et al. 2018), along with the persistent challenges related to safety reporting and emotional support (Habibzadeh et al. 2020; Norouzinia et al. 2024), understanding the prevalence and implications of second victim experience is critical. Exploring the role of safety culture (Alfar et al. 2025; Habibzadeh et al. 2020; Li et al. 2024) and the potential for PTG (Huang et al. 2024; Simms‐Ellis et al. 2025) may inform strategies that not only reduce errors but also support nurses' recovery and development (Scott et al. 2010; Tanabe et al. 2021). Therefore, strengthening these areas could improve the overall quality and safety of patient care in Iran. Accordingly, this study investigates the following hypotheses: (a) patient safety culture is inversely associated with second victim experience; (b) patient safety culture is positively associated with PTG; and (c) second victim experience and PTG are interrelated.

## 2 Methods

### 2.1 Study Design and Settings

This cross‐sectional study was conducted in Hamadan, a city in western Iran, between July and October 2024. Data were collected from Mehr and Imam Hossein hospitals, as well as Beasat, Shahid Beheshti, Farshchian, Sina, and Fatemiyeh hospitals. All nurses employed at these facilities were eligible for participation. The study design and reporting followed the Strengthening the Reporting of Observational Studies in Epidemiology (STROBE) checklist for cross‐sectional studies.

### 2.2 Participants and Sampling

A multi‐stage sampling method was employed. First, hospitals were selected using convenience sampling. Then, participants were chosen from each hospital using proportional stratified random sampling based on the total nursing population and the target sample size.

$$\mathrm{nj}={\left(n/N\right)}^{*}\;\mathrm{Nj}$$

Given the presence of multiple inpatient wards, specific wards were randomly selected for inclusion. Nurses from these wards were recruited through convenience sampling until the predetermined sample size for each hospital was achieved. Eligibility criteria included willingness to participate, a minimum of 1 year of professional nursing experience, and prior experience with a medical error, as self‐reported by the participants. Eligible nurses were required to be employed full‐time, hold at least a bachelor's degree, and have no prior diagnosis of a mental health condition. Exclusion criteria included nurses on leave during data collection, part‐time staff, and those in non‐bedside roles, such as shift coordinators, educators, or managers. Incomplete questionnaires were also excluded. All participants were assured that their self‐reported data would remain confidential.

Based on an expected correlation of *r* = 0.15, a significance level of 0.05, and 85% statistical power, the required sample size to detect the correlation was calculated as 396 participants. To account for an anticipated 10% dropout, the final target sample size was set at 436. Following approval from the ethics committee and hospital authorities, researchers introduced themselves to participants on‐site. Questionnaires were then distributed after fully explaining the study objectives and obtaining informed consent. Participation was entirely voluntary.

### 2.3 Measurements

Data were collected using a demographic and occupational information form, the Second Victim Experience and Support Questionnaire (SVES), the Post‐Traumatic Growth Inventory (PTGI), and the Hospital Survey on Patient Safety Culture (HSOPSC). All responses were self‐reported.

#### 2.3.1 Demographic and Occupational Information

Participants provided demographic and occupational information, including age, gender, marital status, educational level, hospital of employment, years of experience at the current hospital, and average weekly overtime hours. Respondents reported these details based on their typical work conditions over the preceding 6 months.

#### 2.3.2 The Second Victim Experience and Support Questionnaire

The SVES, developed by Burlison et al. (2017), consists of 36 items grouped into nine dimensions: psychological distress, physical distress, colleague support, supervisor support, institutional support, non‐work‐related support, professional self‐efficacy, turnover intention, and absenteeism (Burlison et al. 2017). Responses are rated on a five‐point Likert scale from 1 (Strongly Disagree) to 5 (Strongly Agree), yielding a total score ranging from 36 (minimal second victim experience) to 180 (maximum second victim experience).

The SVES comprises three main sections. The first section assesses healthcare providers' experiences with unintended patient safety events, including direct errors (e.g., medication or transfusion mistakes, incorrect equipment settings, procedural oversights) and near‐miss incidents intercepted before causing harm. The second section evaluates the support received from colleagues and the organisation following such events. The third section examines participants' openness to organisational support and measures two key outcomes—absenteeism and turnover intention—through four specific items (Ajoudani et al. 2021). In the original validation study, Burlison et al. reported internal consistency values ranging from *α* = 0.61 to 0.89 across subscales (Burlison et al. 2017). The Persian version of the SVES was translated and validated by Ajoudani et al. (2021), confirming acceptable validity and reliability in the Iranian context (Ajoudani et al. 2021). More recently, Sharif‐Nia and Hanifi (2023) demonstrated strong psychometric properties of the SVES among Iranian nurses, reporting a Cronbach's alpha of 0.70 for the full scale, with composite and maximal reliability indices exceeding 0.70 (Sharif‐Nia and Hanifi 2023).

#### 2.3.3 Post‐Traumatic Growth Inventory (PTGI)

The Post‐Traumatic Growth Inventory (PTGI), developed by Tedeschi and Calhoun (1996), is a self‐report instrument designed to assess positive psychological changes following traumatic experiences (Tedeschi and Calhoun 1996). The inventory includes 21 items rated on a six‐point Likert scale, ranging from 0 (“I did not experience this change as a result of my crisis”) to 5 (“I experienced this change to a very great degree as a result of my crisis”). Total scores range from 0 to 105, with higher scores indicating greater levels of post‐traumatic growth. The PTGI is structured into five factors: Relating to Others, New Possibilities, Personal Strength, Spiritual Change, and Appreciation of Life. In the original validation study, Tedeschi and Calhoun (1996) reported strong correlations between the subscale scores and the total score (*r* = 0.88, *p* < 0.05), along with excellent internal consistency for the full scale (Cronbach's *α* = 0.92) (Tedeschi and Calhoun 1996). Heidarzadeh et al. (2018) evaluated the Persian version of the PTGI and confirmed its validity, reporting a Cronbach's *α* of 0.87 for the overall scale. Subscale reliability coefficients ranged from 0.57 to 0.77 (Heidarzadeh et al. 2018).

#### 2.3.4 Patient Safety Culture

Patient safety culture was assessed using the validated Persian version of the Hospital Survey on Patient Safety Culture (HSOPSC). This instrument contains 42 items rated on a five‐point Likert scale, from 1 (“Strongly Disagree”) to 5 (“Strongly Agree”). The survey evaluates multiple dimensions of patient safety culture, and an overall score is obtained by summing responses across all items, with higher scores reflecting a stronger safety culture. The reliability and validity of the Persian version of the HSOPSC have been supported by previous research. Habibzadeh et al. (2020) reported a Cronbach's *α* of 0.80 for this version (Habibzadeh et al. 2020). In the current study, the scale demonstrated good internal consistency, with a Cronbach's *α* of 0.84.

### 2.4 Data Analysis

All statistical analyses were conducted using SPSS software (version 25). Continuous variables were reported as mean ± standard deviation, and categorical variables were presented as frequency (%). Normality of the data was assessed using the Kolmogorov–Smirnov test. Differences between groups were examined using independent‐samples *t*‐tests for comparisons involving two groups and one‐way ANOVA for comparisons involving more than two groups. Bivariate correlations among continuous variables were evaluated using Pearson's correlation coefficient. To identify predictors of the second‐victim experience, multiple linear regression analysis was performed, adjusting for gender and work hours as potential confounders. In the regression results, B denotes the unstandardised coefficient, and CI denotes the 95% confidence interval for B. *R* ^2^ indicates the proportion of variance explained by the model. A *p*‐value of less than 0.05 is considered statistically significant.

## 3 Results

Of the 436 participants recruited, 435 completed all questionnaires, yielding a completion rate of 98.8%. One participant (0.2%) was excluded due to incomplete data, and the remaining 435 cases had complete responses for all items. Most participants were female (70.3%, *n* = 306) and married (58.2%, *n* = 253). The mean age of participants was 33.08 ± 7.32 years. The majority of nurses held a bachelor's degree (91.5%) and had more than 10 years of work experience (38.16%). In addition, most nurses (94.9%, *n* = 413) worked more than 30 h per week. Regarding workplace distribution, the largest proportion of nurses (28.5%, *n* = 124) were employed at Besat Hospital. This group represented the largest part of the sample (Table 1). The mean scores for patient safety culture, second‐victim experience, and PTG were 132.31 ± 16.13, 111.72 ± 19.47, and 59.05 ± 21.96, respectively (Table 2).

**TABLE 1:** Demographic and occupational characteristics of the nurses (*N* = 435).

	*n*	%
Gender		
Female	306	70.3
Male	129	29.7
Marital status		
Single	182	41.8
Married	253	58.2
Age (*R*:22–54; *M*:33.08; SD: 7.32)		
≤ 30 years	215	49.43
≥ 31 years	220	50.57
Education		
Bachelor	398	91.5
Master	34	7.8
Ph.D	3	0.7
Tenure in hospital (*R*:1–29; *M*: 8.66; SD: 6.76)		
< 2 years	10	2.30
2–5 years	152	34.94
5–10 years	107	24.60
> 10 years	166	38.16
Hospital		
Farshchian	65	14.9
Besat	124	28.5
Beheshti	70	16.1
Sina	47	10.8
Fatemieh	33	7.6
Emam Hossein	63	14.5
Mehr	33	7.6
Work‐hours		
< 30 h	22	5.1
≥ 30 h	413	94.9

Abbreviations: %, percentage; mean ± SD, mean plus or minus standard deviation; *n*, frequency; *R*, range.

**TABLE 2:** Mean scores and standard deviations of patient safety culture, second victim experience, and post‐traumatic growth (*N* = 435).

	Max	Min	Mean ± SD
Safety culture	210	61	132.31 ± 16.12
Second victim	167	36	111.72 ± 19.47
Post‐traumatic growth	105	10	59.05 ± 21.96

Abbreviations: Max, maximum score; mean ± SD, mean plus or minus standard deviation; Min, minimum score.

Patient safety culture showed a statistically significant negative correlation with SVE (r = −0.303, *p* < 0.001), indicating that a stronger patient safety culture was associated with lower levels of second victim experience. In addition, a statistically significant positive correlation was observed between patient safety culture and PTG (*r* = 0.330, *p* < 0.001), suggesting that greater patient safety culture is linked to higher PTG levels (Table 3).

**TABLE 3:** Pearson correlation coefficients among patient safety culture, second victim experience, and post‐traumatic growth (*N* = 435).

	Safety culture	Second victim	Post‐traumatic growth
Safety culture	1.00	—	—
Second victim experience	*r* = −0.303 *p* < 0.001	1.00	—
Post‐traumatic growth	*r* = 0.330 *p* < 0.001	*r* = −0.041 *p* = 0.395	1.00

Abbreviations: *r*, Pearson correlation coefficient; *p*, *p*‐value.

Data analysis revealed significant differences in SVE scores according to gender, hospital, and overtime (*p* < 0.05). Additionally, PTG scores differed significantly by hospital (*p* < 0.05). However, no significant associations were observed between patient safety culture scores and any demographic variables (*p* > 0.05) (Table 4).

**TABLE 4:** Association between safety culture, second victim experiences, and post‐traumatic growth with demographic characteristics of study participants (*N* = 435).

	Safety culture		Second victim experiences		Post‐traumatic growth	
	Mean (SD)	*p*	Mean (SD)	*p*	Mean (SD)	*p*
Gender						
Male	134.53 (17.10)	0.062	108.05 (18.98)	0.010	59.34 (21.85)	0.854
Female	131.37 (15.63)		113.27 (19.50)		58.92 (22.03)	
Marital status						
Single	131.96 (15.62)	0.703	113.21 (20.17)	0.174	57.5 (21.34)	0.212
Married	132.56 (16.50)		110.64 (18.93)		60.16 (22.36)	
Age						
≤ 30 years	131.43 (14.98)	0.262	112.30 (20.45)	0.541	58.19 (21.71)	0.420
≥ 31 years	133.17 (17.16)		111.15 (18.50)		59.89 (22.21)	
Education						
Bachelor	132.00 (15.74)	0.227	111.83 (18.89)	0.994	58.85 (21.76)	0.547
Master	134.73 (20.12)		110.82 (25.14)		60.11 (24.98)	
Ph.D	145.66 (13.20)		106.66 (31.37)		72.33 (3.21)	
Tenure in the hospital						
< 2 years	128.3 (10.85)	0.844	110.9 (20.87)	0.939	57.7 (27.39)	0.924
2–5 years	132.38 (13.87)		112.06 (19.40)		58.36 (21.99)	
5–10 years	131.89 (16.32)		110.72 (20.51)		58.77 (21.99)	
> 10 years	132.76 (18.14)		112.10 (18.92)		59.93 (21.73)	
Hospitals in Hamadan						
Farshchian	128.81 (20.33)	0.074	114.15 (19.99)	0.045	61.93 (21.86)	*p* < 0.001
Besat	131.5 (14.26)		115.86 (19.33)		56.65 (20.49)	
Beheshti	134.98 (18.29)		108.71 (21.75)		40.07 (24.09)	
Sina	128.23 (12.74)		108.29 (18.90)		62.89 (19.67)	
Fatemieh	139.66 (22.12)		106.96 (21.04)		64.42 (26.57)	
Emam Hossein	133.76 (10.72)		109.20 (16.43)		65.04 (19.09)	
Mehr	132.30 (11.10)		112.21 (16.03)		61.24 (19.08)	
Work‐hours						
< 30 h	133.09 (11.93)	0.817	98.18 (19.87)	*p* < 0.001	58.81 (27.42)	0.959
≥ 30 h	132.27 (16.33)		112.44 (19.21)		59.06 (21.67)	

*Note:* mean (SD), mean and standard deviation; significance level *p* < 0.05.

Multiple linear regression analysis indicated that gender was significantly associated with SVE (*p* = 0.026, 95% CI [0.50, 8.07]), with female nurses reporting higher SVE scores than their male counterparts. In addition, longer work hours were significantly associated with higher levels of SVE (*p* < 0.001, 95% CI [6.400, 22.107]), with participants working more than 30 h per week experiencing more SVE. Furthermore, higher perceptions of patient safety culture were significantly associated with lower SVE scores (*p* < 0.001, 95% CI [−0.491, −0.264]). No significant association was found between SVE and PTG (*p* > 0.05, 95% CI [−0.027, 0.138]). The overall regression model was statistically significant, accounting for approximately 13% of the variance in SVE scores (*R* ^2^ = 0.130, Adjusted *R* ^2^ = 0.122) (Table 5).

**TABLE 5:** Multiple linear regression analysis of factors associated with second victim experience scores (*N* = 435).

Variable	*B*	Std. error	*t*	*p*	95% CI for *B*	Summary of the multiple linear regression model
Gender (Male: reference)	4.28	1.92	2.22	0.026	(0.50, 8.07)	*R* ^2^ = 0.130 Adjusted *R* ^2^ = 0.122 Std. error of estimate = 18.25
Work‐hours (< 30 h: reference)	14.25	3.99	3.57	< 0.001	(6.400, 22.107)	
Safety culture	−0.37	0.05	−6.55	< 0.001	(−0.491, −0.264)	
Post‐traumatic growth	0.05	0.042	1.32	0.187	(−0.027, 0.138)	
Constant	156.18	7.51	20.79	< 0.001	(115.017, 157.483)	

*Note:* women: code 1, men: code 0; work‐hours < 30 h: code 0, work‐hours > 30 h: code 1.

Abbreviations: *B*, unstandardised regression coefficient; Std. error, standard error; *t*, *t*‐statistic; *p*, *p*‐value; CI, 95% confidence interval.

## 4 Discussion

In this study, nurses reported an average level of patient safety culture, elevated levels of SVE, and an average level of PTG. A significant negative correlation was observed between patient safety culture and SVE, whereas a significant positive correlation was found between patient safety culture and PTG. Female nurses and those working more than 30 h per week reported significantly higher SVE scores. Regression analysis further indicated that a higher patient safety culture was associated with lower SVE levels. However, no significant association was observed between SVE and PTG. The final regression model accounted for 13% of the variance in SVE.

The present results, showing an average patient safety culture, high SVE, and moderate PTG, align with findings from both Iranian and international contexts. For instance, in Iran, patient safety culture among nurses in teaching hospitals is typically moderate, indicating a need for improvement (Ebrahimabadi et al. 2022). Similarly, elevated SVE—often accompanied by considerable psychological distress following adverse events—has been widely reported in countries such as South Korea and China (Lee and Lee 2025; Tang, Xie, et al. 2024). This consistency across contexts underscores the global prevalence of the second‐victim phenomenon (Ajoudani et al. 2021).

Regarding PTG, the findings align with evidence indicating that growth following trauma is neither automatic nor uniform and is influenced by factors such as social support, coping strategies, and organisational culture (Wang et al. 2024). Moderate PTG levels suggest that some nurses are able to find positive meaning after trauma, but broader systemic support is needed to enhance this outcome (27). Overall, the moderate patient safety culture indicates a system that is neither weak nor strong, contributing both protective and risk factors to nurse well‐being (Habibzadeh et al. 2020). The high prevalence of SVE highlights the need for improved organisational support, effective leadership, and comprehensive education to mitigate psychological harm. Strengthening the safety culture and providing targeted psychological support are therefore essential steps to enhance nurse well‐being and, in turn, improve patient outcomes (Busch et al. 2021).

### 4.1 Association Between Patient Safety Culture and SVE

The significant negative association between patient safety culture and SVE is consistent with prior research indicating that environments perceived as supportive of safety are linked to lower levels of distress following adverse patient events. This finding supports our first hypothesis, which proposed an inverse relationship between these variables. A positive safety culture fosters organisational support and trust, which can potentially reduce the emotional burden and negative work outcomes. Nurses with stronger perceptions of patient safety culture tend to exhibit fewer adverse psychological responses and feel more supported after adverse events (Eslami et al. 2022; Habibzadeh et al. 2020; Li et al. 2024). However, evidence suggests that although a stronger safety culture enhances perceptions of organisational support, it may not directly alleviate distress (Eslami et al. 2022). Moreover, improvements in patient safety culture have been linked to enhanced support systems that help mitigate negative work outcomes such as absenteeism and turnover. Such improvements also promote trust, encourage non‐punitive responses, and strengthen teamwork, all of which may buffer the emotional burden associated with adverse events (Mahat et al. 2025; Mohd Kamaruzaman et al. 2022).

### 4.2 Association Between Patient Safety Culture and PTG

Our second hypothesis, proposing a direct relationship between patient safety culture and PTG, was supported. A significant positive correlation was observed, indicating that nurses working in environments with a stronger patient safety culture reported higher levels of positive psychological growth following traumatic incidents (Mahat et al. 2025; Shomalinasab et al. 2023; Talebi et al. 2021). This suggests that a supportive and just culture not only alleviates distress but also fosters conditions conducive to growth. By promoting teamwork, continuous learning, and institutional support, a strong patient safety culture may provide a context in which nurses can process difficult events, find new meaning, and develop enhanced professional and personal strengths (Li et al. 2024; Mohd Kamaruzaman et al. 2022; Scott et al. 2010).

Central to these processes are organisational leadership and peer support. Leaders who encourage open communication and prioritise emotional well‐being help normalise distress and promote constructive reflection, both essential for PTG (Simms‐Ellis et al. 2025; Willis et al. 2019). Educational interventions integrating resilience training and promoting a just culture further strengthen this process by equipping nurses with the cognitive and emotional tools necessary to transform trauma into growth opportunities (Chen et al. 2021; Tang, Yobas, et al. 2024). Overall, the synergy between a positive patient safety culture and PTG functions not merely to mitigate negative psychological outcomes but as a proactive foundation for sustained well‐being and professional development among nursing staff in high‐stress healthcare environments.

### 4.3 Lack of Association Between SVE and PTG

While patient safety culture was associated with lower levels of SVE and higher PTG, the third hypothesis—predicting a significant association between SVE and PTG—was not supported. This non‐significant finding is notable and may be influenced by factors that warrant further exploration, particularly those related to the broader organisational and social context of nursing practice in Iran.

PTG is a complex, dynamic process dependent on effective coping strategies, social support, and organisational culture. Cross‐sectional study designs limit the ability to capture this time‐dependent growth, and participants may have been in the early stages of coping, with PTG not yet fully developed (Chen et al. 2021; Huang et al. 2024). Additionally, maladaptive coping strategies, such as avoidance or emotional suppression—often reinforced by limited psychological and institutional support—can impede PTG even in the presence of distress (Mahat et al. 2025; Tang, Xie, et al. 2024).

When interpreting these findings, it is essential to consider the distinctive sociocultural characteristics of the Iranian healthcare system. Hierarchical organisational structures, fear of blame, and the stigma surrounding error disclosure often discourage open communication about mistakes (Hannani et al. 2020; Hashemi et al. 2012). A qualitative study in an Iranian teaching hospital identified fear of legal repercussions, ineffective reporting mechanisms, and a punitive workplace culture as major barriers to incident reporting, leading to mistrust and concealment (Askarian et al. 2020). Broader national evidence also indicates that patient safety culture in Iran remains at a moderate level, with ongoing challenges in teamwork and non‐punitive responses to errors (Habibzadeh et al. 2020; Matin et al. 2018). These organisational and cultural factors may intensify nurses' psychological strain and limit opportunities for constructive adaptation and PTG. Additionally, prevailing sociocultural norms that discourage emotional vulnerability—particularly among women—further reduce the likelihood of emotional disclosure and reflective growth following adverse events (Eslami et al. 2022; Shahbazzadeh et al. 2025).

Taken together, these findings suggest that the relationship between SVE and PTG among Iranian nurses is influenced not only by organisational and psychological factors but also by broader social norms and institutional practices. Consequently, interventions designed to enhance patient safety culture and support second victims should incorporate culturally sensitive approaches that promote openness, emotional expression, and reflective dialogue, alongside systemic organisational improvements (Järvisalo et al. 2024; Li et al. 2025).

### 4.4 Explaining Higher SVE in Female Nurses and Those Working Over 30 Hours

The higher levels of SVE reported by female nurses in this study can be understood within the broader sociocultural and organisational context of Iran. Iranian gender norms emphasize caregiving, emotional sensitivity, and self‐sacrifice as expected characteristics of women, which increase emotional labor in nursing and heighten vulnerability following patient safety incidents. Zamanzadeh et al. (2013) explain that nursing in Iran is widely regarded as a feminine profession, associated with altruism and caregiving, resulting in men often experiencing stigma and isolation within this predominantly female field. Women's social roles amplify their emotional involvement, making them more susceptible to distress after patient safety events (Zamanzadeh et al. 2013). Furthermore, the persistent gendered image of nursing in Iran, exemplified by the barriers faced by male nursing students (Hosseini et al. 2022), reinforces traditional expectations for female nurses, thereby intensifying their emotional labour. Alinejad Mofrad et al. (2024) further note that traditional cultural and religious expectations about gender profoundly shape female nurses' workplace challenges, particularly when caring for male patients, thereby increasing psychological burden and the likelihood of emotional suppression as a coping strategy (Alinejad Mofrad et al. 2024).

These gendered expectations intersect with organisational factors. Hierarchical structures and the predominantly punitive error‐reporting climate in Iranian hospitals may disproportionately affect female nurses, who often hold lower organisational authority and therefore perceive reduced psychological safety when attempting to discuss mistakes. Previous Iranian studies have documented fear of blame, punitive management practices, and limited support for error reporting, all of which contribute to higher psychological strain among nurses (Habibzadeh et al. 2020; Hashemi et al. 2012; Matin et al. 2018). In such environments, female nurses may rely more frequently on avoidance‐based or emotion‐suppressing coping strategies—patterns strongly associated with heightened second victim distress (Quillivan et al. 2016).

Long working hours further intensify these dynamics. Female nurses in Iran commonly manage significant domestic responsibilities alongside extended clinical shifts, resulting in limited opportunities for emotional recovery and greater cumulative fatigue. This dual burden, shaped by both cultural norms and organisational demands, increases vulnerability to second victim distress (Heidarzadeh et al. 2018; Shahbazzadeh et al. 2025).

Collectively, these cultural, organisational, and gender‐related factors provide a coherent and contextually grounded explanation for the elevated levels of second victim experience among female nurses. Addressing these disparities requires culturally informed, gender‐responsive interventions that enhance psychological safety, reduce punitive workplace practices, and expand access to peer and organisational support systems (Eslami et al. 2022; Mokhtari et al. 2018; Shahbazzadeh et al. 2025).

### 4.5 Interpretation of the Regression Model Explaining 13% Variance

Although the regression model explained only 13% of the variance in the dependent variable, this outcome is consistent with extensive organizational and psychosocial research demonstrating that complex human experiences—such as those of second victims in healthcare—emerge from multiple interacting influences rather than single predictive factors. Similar levels of explained variance have been reported in studies of psychological distress and responses to adverse events, affirming that such phenomena cannot be fully accounted for by linear or single‐domain models (Shahbazzadeh et al. 2025; Zheng et al. 2025). Contemporary literature highlights that psychosocial outcomes related to workplace distress are shaped by the dynamic interplay of organisational culture, peer and managerial support, individual resilience, workload, and leadership style (High and Forest 2025; Mira et al. 2025). Although patient safety culture is an important determinant, additional factors—including incident management practices, the availability of social support, individual coping strategies, and the presence of a just culture—substantially influence second victim experiences (Quillivan et al. 2016). The current findings align with these multidimensional frameworks, reflecting the inherently complex nature of workplace behaviour in which organisational outcomes emerge from the interplay of formal structures, leadership practices, team dynamics, and individual attributes. This complexity underscores the need for comprehensive, system‐level interventions aimed at fostering non‐punitive, psychologically safe, and supportive environments for healthcare professionals (Busch et al. 2021; Mira et al. 2025). Effective strategies should integrate organisational‐level reforms—such as strengthening safety culture and enhancing leadership competence—with individual‐level supports, including resilience training and access to psychological counselling (Simms‐Ellis et al. 2025; Willis et al. 2019).

Moreover, the relatively low proportion of explained variance in the regression model underscores the complex and multifaceted nature of second victim experiences in healthcare environments. These experiences arise from a dynamic interplay of psychological, organisational, and contextual factors that are difficult to fully capture through quantitative models alone. In this study, the use of a combined convenience and proportional stratified random sampling method—although practical across multiple hospitals—may have contributed to the limited explanatory power of the model. Nevertheless, this methodological consideration does not negate the inherent challenges of modelling such intricate human experiences (Foji et al. 2023; Mahat et al. 2025). Accordingly, future research should incorporate longitudinal and mixed‐methods designs, along with more comprehensive and randomised sampling strategies, to better elucidate the multifactorial determinants of second victim phenomena. Context‐sensitive investigations remain essential for deepening our understanding of these complex dynamics and for informing the development of tailored, sustainable interventions across diverse healthcare settings (Busch et al. 2021). Ultimately, the findings highlight the importance of system‐level approaches that cultivate psychologically safe, supportive, and non‐punitive work environments—conditions critical for healthcare professionals navigating the challenges inherent to second victim experiences.

### 4.6 Strengths and Limitations of This Study

This study has a few limitations that should be considered when interpreting the findings. First, the cross‐sectional design prevents causal inferences; thus, the associations identified among patient safety culture, second victim experience, and post‐traumatic growth should be interpreted as correlational rather than causal. Longitudinal research is needed to establish temporal relationships and determine causality. Second, reliance on self‐reported data—particularly regarding experiences with medical errors—may introduce social desirability and recall biases. Nurses may have underreported negative or emotionally distressing events due to fear of blame or stigma, potentially resulting in an underestimation of second victim experience.

Additionally, although the combined convenience and proportional stratified random sampling strategy was practical for recruiting participants across multiple hospitals and wards, it inherently carries the risk of selection bias. Nurses who were more accessible, available, or motivated to participate may be overrepresented, which may affect the sample's representativeness and limit generalisability to the broader nursing population. Moreover, data collection was restricted to public hospitals in Iran, which may not reflect the experiences of nurses in private hospitals, non‐hospital healthcare settings, or in different cultural and organisational contexts. Therefore, caution is warranted when extending these findings beyond the study environment.

Despite these limitations, the study offers notable strengths. The use of validated and culturally adapted instruments to assess patient safety culture and second victim experience enhances the reliability and contextual relevance of the findings. The results provide meaningful insights into the Iranian healthcare system and contribute to ongoing efforts to strengthen patient safety and support nurses following adverse events. Future research should employ longitudinal or experimental designs to establish causal relationships, integrate self‐reported data with objective indicators to minimise bias, and adopt fully randomised sampling techniques to improve representativeness. Including a broader range of healthcare settings and organisational contexts will further enhance the generalisability and applicability of future findings.

### 4.7 Nursing implications—integrated with theory

1. The findings of this study offer meaningful guidance for nursing leaders seeking to translate empirical evidence into organisational improvements. Grounded in organisational change theory and the principles of a just culture, the following recommendations emphasise both structural and cultural reforms aimed at reducing second victim experiences and fostering PTG among nurses.
2. Establish formal support programs for second victims:
  Developing structured support mechanisms represents a direct application of just culture principles, which advocate for supportive, non‐punitive responses to staff involved in adverse events. These programs, staffed by trained peer supporters, constitute a targeted organisational change intervention that institutionalises psychological safety by ensuring timely, empathetic, and consistent support, thereby mitigating emotional harm and facilitating recovery.
3. Promote a non‐punitive error reporting culture:
  A non‐punitive reporting environment is central to the just culture framework, which shifts responsibility from individual blame toward system‐level learning. Nursing leadership can advance this cultural shift by implementing anonymous, voluntary reporting systems and modelling transparent communication. From an organisational change perspective, these actions promote collective learning, reduce fear of reprisal, and serve as catalysts for adaptive changes that reinforce a stronger patient safety culture.
4. Integrate resilience and PTG training into continuing education:
  Embedding resilience and PTG training into ongoing professional development reflects a strategic, long‐term change management initiative. Consistent with frameworks such as Kotter's 8‐Step Process, these educational programs help reinforce new attitudes and behaviours that support a more resilient organisational culture. Providing nurses with practical tools to process adverse events aligns with the core tenets of a just culture by enabling staff to reframe trauma and cultivate growth.
5. Implement workload management policies:
  Workload regulation represents a critical structural change necessary to support sustainable behavioural and cultural transformation. Policies designed to limit mandatory overtime and improve nurse‐to‐patient ratios address a known risk factor for SVE. From an organisational change theory standpoint, these measures signal leadership's commitment to staff well‐being and create the conditions necessary for a genuine, just and learning culture—one that seeks system‐level solutions rather than expecting individual resilience to compensate for structural deficiencies.

## 5 Conclusion

Patient safety culture was significantly associated with a reduction in nurses' second victim experiences and an increase in PTG. Female nurses and those working more than 30 h per week demonstrated greater vulnerability to second victimisation, highlighting the need for targeted organisational attention. These findings emphasise the importance of implementing interventions that strengthen safety culture—such as promoting non‐punitive error reporting and establishing structured support programs—to help alleviate psychological distress and enhance resilience among nursing staff. Addressing workload issues is also critical, as excessive work hours may heighten the risk of second victim experiences. Overall, reinforcing patient safety culture and providing tailored support to at‐risk groups may contribute to improved nurse well‐being and enhanced patient safety outcomes. Ultimately, fostering such environments is not only an ethical imperative but also a strategic necessity for building sustainable and high‐reliability healthcare systems.

## Author Contributions

**Hamidreza Siavashi:** conceptualisation, methodology, data curation, writing – original draft, formal analysis, visualisation, investigation, supervision, writing – review and editing, validation. **Ali Safdari:** conceptualisation, methodology, data curation, writing – original draft, formal analysis, visualisation, investigation, supervision, writing – review and editing, validation. **Maryam Farhadian:** conceptualisation, methodology, formal analysis, visualisation, investigation, validation. **Maryam Maddineshat:** conceptualisation, methodology, data curation, writing – original draft, formal analysis, visualisation, investigation, software development, writing – review and editing.

## Funding

The Vice‐Chancellor for Research at Hamadan University of Medical Sciences has provided financial support for this project in 2024 (Grant agreement No. 140304122980).

## Ethics Statement

Participation in this study was voluntary, and all participants were fully informed of the study's purpose, procedures, potential risks and benefits, and confidentiality safeguards. Written informed consent was obtained from each participant, emphasising their right to withdraw at any time without penalty or impact on their employment status. To ensure anonymity, all data were de‐identified using unique codes. The study protocol was approved by the Research Ethics Committee of Hamadan University of Medical Sciences (Approval ID: IR.UMSHA.REC.1403.219). The study was conducted in accordance with the principles of the Declaration of Helsinki.

## Conflicts of Interest

The authors declare no conflicts of interest.

## Data Availability

The datasets produced and scrutinised in this research are not publicly accessible due to the sensitive nature of the information contained within the raw data, which could potentially breach participants' privacy. However, interested parties may request access to the datasets from the corresponding author.
